# Whole Blood Transcriptome Analysis in Children with Sickle Cell Anemia

**DOI:** 10.3389/fgene.2021.737741

**Published:** 2022-01-13

**Authors:** Beatrice E. Gee, Andrea Pearson, Iris Buchanan-Perry, Roger P. Simon, David R. Archer, Robert Meller

**Affiliations:** ^1^ Department of Pediatrics, Morehouse School of Medicine, Atlanta, GA, United States; ^2^ Morehouse School of Medicine, Cardiovascular Research Institute, Atlanta, GA, United States; ^3^ Children’s Healthcare of Atlanta, Atlanta, GA, United States; ^4^ Translational Stroke Program, Neuroscience Institute, Morehouse School of Medicine, Atlanta, GA, United States; ^5^ Grady Memorial Hospital, Atlanta, GA, United States; ^6^ Department of Neurology, Morehouse School of Medicine, Atlanta, GA, United States; ^7^ Aflac Cancer and Blood Disorders Center of Emory University and Children’s Healthcare of Atlanta, Atlanta, GA, United States

**Keywords:** sickle cell disease, RNA-sequencing, transcriptome, eQTL analysis, pathway analysis

## Abstract

Whole transcriptome RNA-sequencing was performed to quantify RNA expression changes in whole blood samples collected from steady state sickle cell anemia (SCA) and control subjects. Pediatric SCA and control subjects were recruited from Atlanta (GA)—based hospital(s) systems and consented for RNA sequencing. RNA sequencing was performed on an Ion Torrent S5 sequencer, using the Ion Total RNA-seq v2 protocol. Data were aligned to the hg19 reference genome and analyzed in the Partek Genomics studio package (v7.0). 223 genes were differentially expressed between SCA and controls (± 1.5 fold change FDR *p* < 0.001) and 441 genes show differential transcript expression (± 1.5 fold FDR *p* < 0.001). Differentially expressed RNA are enriched for hemoglobin associated genes and ubiquitin-proteasome pathway genes. Further analysis shows higher gamma globin gene expression in SCA (33-fold HBG1 and 49-fold HBG2, both FDR *p* < 0.05), which did not correlate with hemoglobin F protein levels. eQTL analysis identified SNPs in novel non-coding RNA RYR2 gene as having a potential regulatory role in HBG1 and HBG2 expression levels. Gene expression correlation identified JHDM1D-AS1(KDM7A-DT), a non-coding RNA associated with angiogenesis, enhanced GATA1 and decreased JAK-STAT signaling to correlate with HBG1 and HBG2 mRNA levels. These data suggest novel regulatory mechanisms for fetal hemoglobin regulation, which may offer innovative therapeutic approaches for SCA.

## Introduction

Sickle cell anemia (SCA) is a genetically inherited blood disorder. Homozygous hemoglobin SS is characterized by a single point mutation (T to A) of the beta hemoglobin gene (Hbb), resulting in a non-conservative protein mutation at the sixth codon (Glu to Val) (Pauling et al., 1949; Ingram, 1958). This mutation results in polymerization of the hemoglobin molecules under low oxygen conditions, and the classic sickling morphology of red blood cells (Scriver and Waugh, 1930). Despite a single genetic mutation being the root cause, there is considerable heterogeneity between each individual’s disease severity, and predicting who is at heightened risk of vascular complications is challenging. Circulating immune cells are in contact with the vasculature and are involved in cardiovascular diseases. Using these circulating cells as sentinels, one can diagnose various brain insults using RNA expression patterns in peripheral blood, including stroke (Sharp et al., 2007; Sharp et al., 2011; Meller et al., 2016), with high accuracy and the sensitivity to different causes of stroke (Jickling et al., 2011; Jickling et al., 2012; Jickling et al., 2013). A recent study described a link between such peripheral blood transcriptome profiles with patient mortality in sickle disease (Desai et al., 2017). To date, published studies have used microarray technology to assess known gene expression profiles in sickle cell disease (Jison et al., 2004; Raghavachari et al., 2007; Raghavachari et al., 2009; Raghavachari et al., 2012; Desai et al., 2017). Based on the number of inflammatory derangements described in sickle cell disease [for a review see (Balandya et al., 2016)], we expect that, in comparison to people without SCA, circulating immune cells from SCA subjects will express distinctly different genes that mediate the disease phenotype. In order to further study the pathologic mechanisms of SCA, and identify potential therapeutic targets, we investigated peripheral blood whole transcriptome profiling in pediatric patients with SCA.

## Methods

### Study Approval

All procedures were reviewed and approved by Institutional Review Boards at Children’s Healthcare of Atlanta and Morehouse School of Medicine (14-125 CHOA). All participants or guardians gave written informed consent prior to their enrollment in the study. Samples were de-identified prior to analysis.

### Participant Recruitment

African American children and young adults (3–21 years old) were recruited from Children’s Healthcare of Atlanta Aflac Sickle Cell Clinics and Morehouse Healthcare Pediatrics Clinic, Atlanta, GA. Subjects with SCA had Hemoglobin SS or S-β^0^-thalassemia, without transfusions in the past 3 months or hydroxyurea therapy in the past 6 months. Healthy controls were self-identified African American children or young adults (3–21 years old), with hemoglobin genotypes AA, AS, AC, or β-thalassemia trait confirmed by electrophoresis (Quest Diagnostics). Exclusions included acute illness, chronic diseases other than SCA, pregnancy, and history of cardiovascular risk factors (BMI >95th %ile for age, high cholesterol or other hyperlipidemia, diabetes, cigarette smoking more than five per day, or hypertension treated with medications). Whole blood (3 ml) was collected in PAXgene RNA tubes. Clinical data were extracted from the medical record included anthropometrics, complete blood count, and medical history.

### RNA-Seq Library Assembly

RNA was extracted from 3 ml samples of blood (stored in Paxgene tubes at −20°C) using the Pre-Analytix RNA extraction kit (Qiagen). RNA-seq libraries were constructed using the Ion Total RNA-Seq Kit v2 (ThermoFisher Scientific) with 500 ng total RNA as starting material (not globin depleted). RNA was sheared using RNaseIII, ligated to adapters, and reverse transcribed with Super Script^TM^III. cDNA was size selected using Ampure XL beads (Beckman Coulter) and amplified using Platinum™ DNA polymerase (15 cycles) with IonXpress barcode primers (1–16). Libraries were quantified using High Sensitivity DNA Bioanalyzer chips, pooled, and cloned onto sequencing spheres using an Ion OneTouch2. Templated spheres were loaded on Ion 540 chips and sequenced on an Ion Torrent S5 DNA sequencer, using the RNA-Seq analysis plugin. Data were aligned to the hg19 reference genome using STAR and Bowtie2. Resultant Bam data files were uploaded to Partek Genomics Studio software (v 7.0). Gene and transcript expression values were determined using the Ensemble v87 annotation guide for Hg19 (downloaded 01/2018). (Original data has been submitted to dbGAP. For other data request please contact rmeller@MSM.edu).

### Differential Expression Analysis

Gene and transcript expression was calculated as reads per kilobase of transcript per million mapped reads (RPKM) using Partek Genomic Suite (V 7.0). Genes with a fewer than 10 aligned reads in 25% of samples were filtered out. The data were normalized using trimmed mean method. Data were subjected to one-way analysis of variance (2-way ANCOVA) using clinical SCA status as the factor, corrected for age, and sex in the linear model. We did not correct for weight, because one control had that data missing. Data with a 1.5-fold difference in expression levels and passing a false discovery rate (FDR) of 0.001 were considered significantly expressed and considered for further analysis. PCA and hierarchical clustering was performed in Partek genomics suite using gene expression values of significantly regulated genes and transcripts. Volcano plot was generated using enhancedVolcano, by importing the ANOVA output data sheet and selecting the contrast specific *p* value and the fold change. Gene Set Ontology Analysis was performed using Partek Genomics suite (v7.0) and ClueGo App for Cytoscape (Bindea et al., 2009). Partek Genomics Suite’s linear support vector machine (SVM) classifiers with shrinking centroids was used to train models for predicting CA diagnosis. Models with a normalized correct rate greater than 85% were then tested using two level cross-validation (full leave one out and groups of 10) to determine accuracy, sensitivity, and specificity.

### Correlation Analysis

Correlation analysis was performed on the normalized rpkm values of HGB1 and HGB2 vs the rest of the transcriptome using Spearman correlation in Partek Gene studio (v 7.0) correlation images were prepared in R using the Hmisc package. Data were initially correlated together, and then for SCA and Control groups separately. Significance was corrected for repeated measures using the Benjamini-Hochberg/Bonferroni correction. An adjusted *p* value of 0.05 was accepted as significant. Data for individual gene pairs were plotted in Graphpad.

### eQTL Analysis

Data were analyzed using MATRIXeQTL in R (Shabalin, 2012). SNP calls were generated and processed in Partek, using the detect nucleotide variation workflow. Data were called if LOR greater or equal to 50, and then filtered for a mean number of reads of 20. Data were annotated with known SNP (dbSNP151) identifiers in Partek. The resultant base call matrix was saved as a text file (available on request from R.Meller) and transferred into R for processing as a numerical matrix using the SNPready package. Gene expression data and co-variates data were also prepared as numeric databases (see attached script). Data were analyzed for SNPS affecting HBG1 and HBG2 expression, using a linear model, correcting for age, sex, and SCA status. Data were plotted using CMplot (Yin et al., 2021). Significant genes were plotted against base calls using Graphpad Prism v6. A script of R based procedures is included in the Supplementary Material.

### Statistics

The target number of subjects enrolled was based on power analysis of previous data (Meller et al., 2016). Thirty subjects in SCA and Control groups would enable detection of over 75% of 1.2-fold differences in gene expression, if the between group differences were comparable to African American adults with or without stroke. Normality, *t*-test, chi square, Mann Whitney U, Wilcoxon signed rank, and Fisher’s exact tests were performed with GraphPad Prism (v6.0). A *p*-value <0.05 was considered significant. Analysis of variance (ANOVA) was performed on TMM normalized RPKM values to determine differential expression using Partek Genomics Suite at a significance cutoff of ± 1.5 fold change and a post-hoc False Discovery Rate (FDR) correction (*p* < 0.001). Principal component analysis and hierarchical clustering were used to assess clustering of the data (gene or transcript expression values). Accuracy, sensitivity, specificity, and area under the curve (AUC) for the prediction models were also determined using Partek Genomics Suite (v 7).

## Results

### Participant Enrollment

We recruited 48 subjects for this study, 31 with SCA and 18 controls. One participant was withdrawn from the study because on subsequent chart review it was determined they were being medicated with hydroxyurea (HU) therapy at the time of blood sample collection (above exclusion criteria). All HU-free SCA subjects were homozygous for the HbSS genotype, based on medical history and confirmed by SNP analysis of the sixth codon of the HBB gene. Patients heterozygous for the sickle cell causing Hbb gene mutation (HbAS) were grouped with the control samples (HbAA). All analysis was performed on mixed sex samples corrected for age and sex (see Table 1). The age of SCA patients was lower than controls, (8 vs. 12 years, *p* = 0.0054 Mann Whitney *U* test: Table 1). Control subjects had a higher average BMI than SCA (20.27 ± 3.6 vs. 16.67 ± 2.7, *p* < 0.001 Student’s t test; Table 1). Average hemoglobin concentration for controls was 13.09 g/dl, compared to 8.8 in SCA (Table 1), and 8.2 in SCA with cerebral arteriopathy (not shown). Average fetal hemoglobin was significantly higher for SCA subjects compared to controls (14.9 ± 7.6% vs. < 1%, *p* < 0.0001, Wilcoxon Signed Rank test; g/dl shown in Table 1). There was no difference in the mean corpuscular volume between controls and SCA (Table 1).

**TABLE 1:** Patient information. Data provided on 46 participants who were sequenced and subjected to further analysis.

Patient data	Control	SCD		*p* value		Test
Age (Yr)	Mean	12.1	8.4	0.0031	**	Mann Whitney *U* test
	SD	3.6	3.8			
Sex	Mean	8	10	0.6193	ns	chi-squared
	Female	10	17	0.7582		Fishers exact test
Weight (kg)	Mean	49.1^a^	28.1	0.0002	***	Student’s t test
	SD	18.5^a^	15.04			
Height (cm)	Mean	152.2	126.5	0.004	***	Student’s t test
	SD	17.6	23.4			
BMI	Mean	20.3	17.6	0.007	***	Mann Whitney *U* test
	SD	3.6	2.8			
Hbb (g/dl)	Mean	13.09	8.83	< 0.0001	****	Student’s t test
	SD	1.04	1.01			
HbF (g/dl)	Mean	0.13	1.3	< 0.0001	****	Wilcoxon Sign Ranked test
	SD	0.01	0.654			
Mean Corpuscular	Mean	86.8	83.3	0.154	ns	Student’s t test
Volume	SD	5.8	9.2			

a denotes data from 16 controls.

### Alignment and Mapping Statistics

**FIGURE 1:**
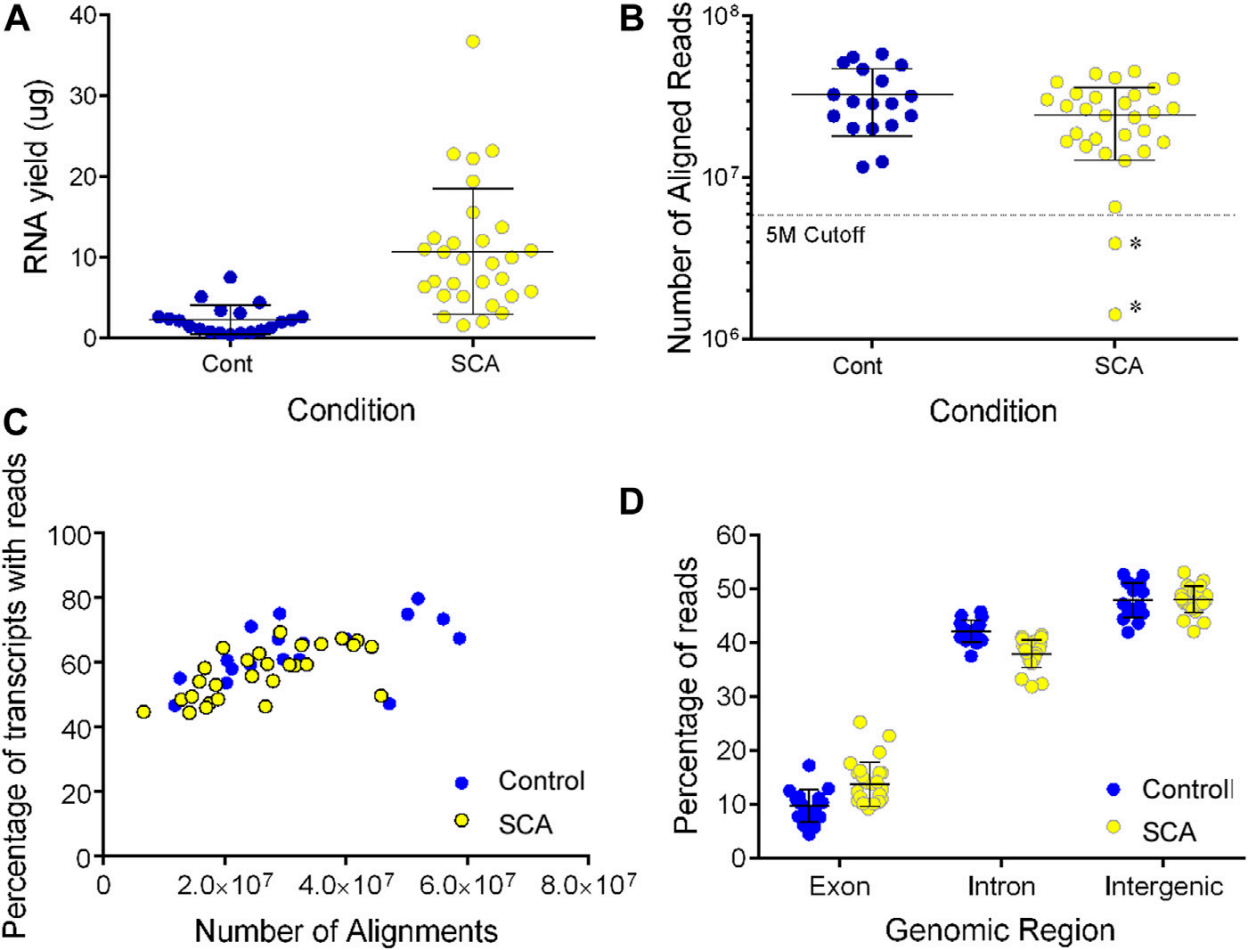
RNA sequencing of SCA(26) and control blood samples (18). **(A)** RNA was extracted from 3 ml whole blood samples using the preAnalytix isolation kit and quantified by absorbance. Yield was calculated as µg in 18 control (blue) and 30 SCA (yellow) samples (Note three samples were excluded from further study. **(B)** Following sequencing, the number of reads aligned to the hg19 reference genome was determined using Partek Genome Studio (V7.0). Samples marked as * show two additional samples below our five million aligned reads cutoff value, and were excluded from further analysis, data were not significantly different (*p* = 0.07; unpaired *t*-test). **(C)** The number of gene transcripts identified in SCA (yellow) and controls (blue) were shown with respect to the depth of sequencing (number of aligned reads). **(D)**. Following alignment and mapping, the number of reads aligning to exonic, intronic, or intergenic genomic regions was determined in Partek genomics studio. Data were analyzed using 1 way ANOVA with post-hoc Sidak’s multiple comparison test subjected to (** denotes *p* < 0.001).

We noted a higher yield of RNA extracted from SCA patients’ blood samples than controls (10.8 vs. 2.3 µg/3 ml blood sample, *p* < 0.001 Student’s t test: Figure 1A). Three samples failed our sequencing QC, either due to poor RNA library with evidence of degradation (1), or low number of reads < 5 million (2: annotated with an * in Figure 1B). These data, and that from the HU treated subject, were excluded from further analysis.

Following alignment, we observed no significant difference in the number of aligned reads between control of SCA patients (32.8 vs. 26.2 million aligned reads, *p* > 0.05; Figure 1B). There was no effect of age on the number of aligned reads (data not shown). The detection of transcripts plateaued to approximately 68% of transcripts in all samples (_1/2_ max at five million reads: Figure 1C). The ratio of exonic, intronic, and intergenic reads was similar to our previous blood transcriptome estimates (Meller et al., 2016; Hardy et al., 2017). When we compared the mapping statistics, we observed a significantly higher mapping of RNA to exonic regions in libraries prepared from SCA subjects compared to those prepared from controls. There was a converse reduction of intronic mapping of reads in SCA-derived libraries compared to control-derived libraries (Figure 1D; both *p* < 0.001 1 way ANOVA with Sidak’s multiple comparison test). There was no difference in intergenic mapping between groups.

### Differential Expression Analysis

**FIGURE 2:**
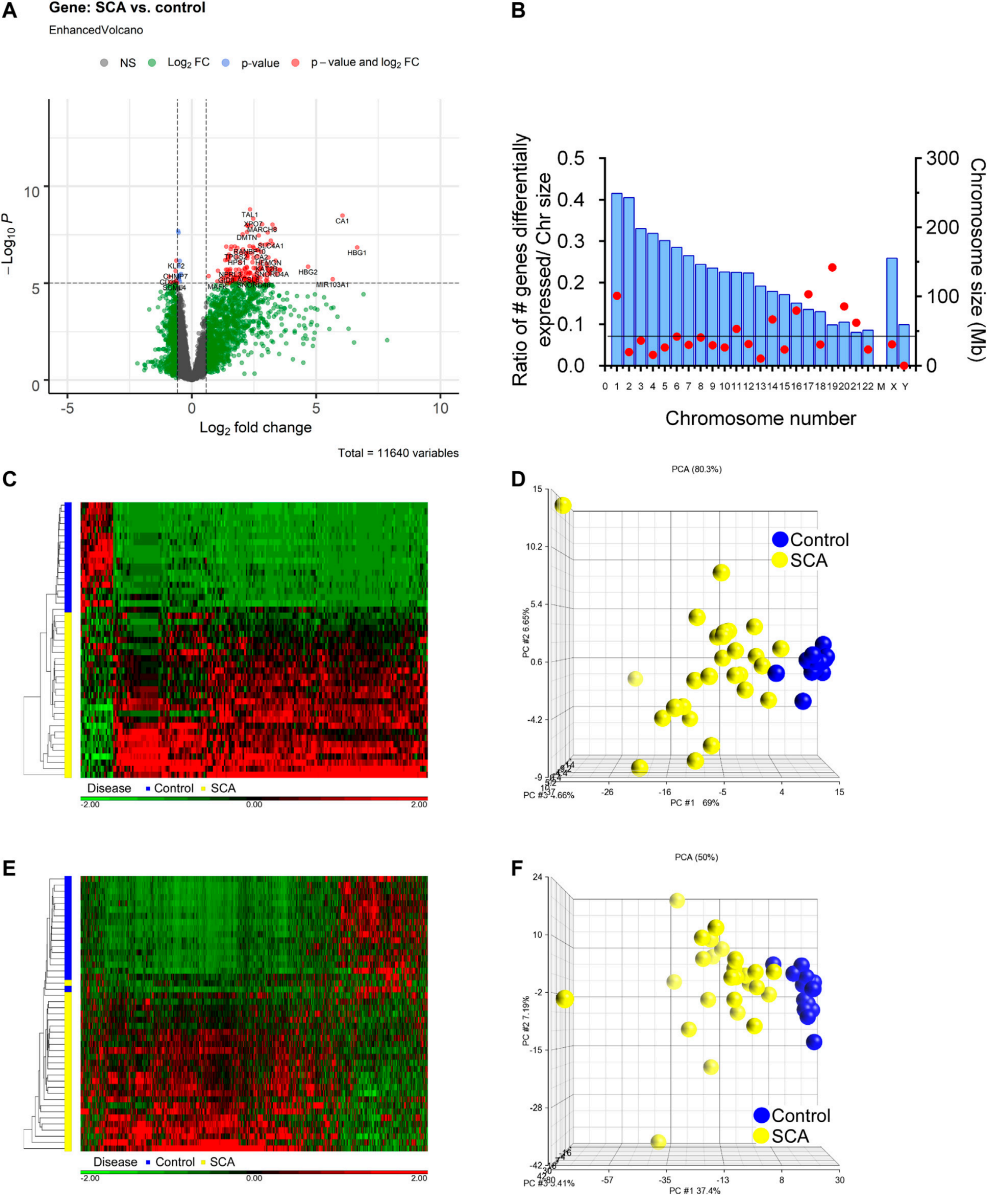
Differential gene expression and transcript splicing in SCA patients. **(A)** Volcano plot showing differentially expressed RNAs, as a measure of fold change and *p*-value. Data in gray denote data with less than 1.5 fold change, or not significantly different [ANCOVA (correcting for age and sex) with adjusted FDR *p* < 0.001]. Data in red show up or down-regulated genes passing fold change and *p* value cutoff. **(B)** Chromosome location of differentially expressed genes. Chromosome size is shown in blue bars. The number of differentially expressed genes were expressed as a ratio with respect to chromosome size. The line denotes expected ratio of all genes and all chromosome sizes. **(C)** Hierarchical cluster of 557 differentially expressed genes Red denotes genes with increased expression, and green denotes decreased expression. On the vertical axis blue and yellow bars denote control and SCA patient samples, respectively. Black denotes patients with CA. **(D)** Principal component analysis of gene expression data shows 73.7% of the variability within the samples is accounted by three principal components (calculated using Partek). Note clustering of SCA (yellow) vs. Controls (blue). **(E)** Hierarchical cluster of differential transcript expression. Red denotes increased transcript splicing, and green denotes decreased splicing. On the vertical axis blue and yellow bars denote control and SCA patient samples, respectively. **(F)** Principal component analysis of differential transcript expression data shows 69.6% of the variability within the samples is accounted by three principal components. Note clustering of SCA (yellow) vs. Controls (blue).

We determined differential gene and transcript expression between SCA and Control groups. Analysis revealed 557 genes with ± 1.5-fold differential expression in SCA patients compared to controls, which was decreased to 223 differentially expressed genes when corrected for age and sex [2-way ANCOVA (correcting for age and sex); FDR *p* < 0.001 (Benjamini-Hochberg)]. Many genes showed higher expression in SCA compared to control (Figure 2A). There was an enrichment of differential expression in Chr1, Chr17, and Chr19, and an underrepresentation of Chr13, Chr21 and ChrY (Figure 2B). The differentially expressed genes were subjected to hierarchical clustering, and principal component analysis (Figures 2C,D). Hierarchical clustering of the samples shows a clear clustering of the controls and the SCA subjects based on gene expression values, with the exception of one SCA patient (red denotes increased expression). Three principal components account for 80.3% of the variability between the samples (Figure 2D). Of note the first principal component accounted for the effect of SCA status.

Analysis of transcript expression revealed 441 transcripts to be differentially expressed (alternatively spliced) in SCA compared to controls (1-way ANCOVA ± 1.5 fold change with FDR *p* < 0.001). These data cluster with respect to SCA or control conditions (Figure 2E). Principal component analysis shows three components account for 50% of the variability (Figure 2F). The differentially expressed transcripts (441) were mapped to genes (223). When we compare the genes with differential expression and the genes with alternative splicing, we find 204 genes overlap 46% of transcripts map to differentially expressed genes, and 91% of DEGs map to alternatively spliced transcripts (not shown). This suggests that most genes with differential expression also show differential transcript usage in SCA. Additionally, multiple genes express more than one alternative spliced transcript. It was noted that multiple hemoglobin-associated genes (HBB, HBD, HBG1, HBG2, and HBM) show differential isoform usage in SCA.

### Functional Analysis of SCA-Regulated Genes

**FIGURE 3:**
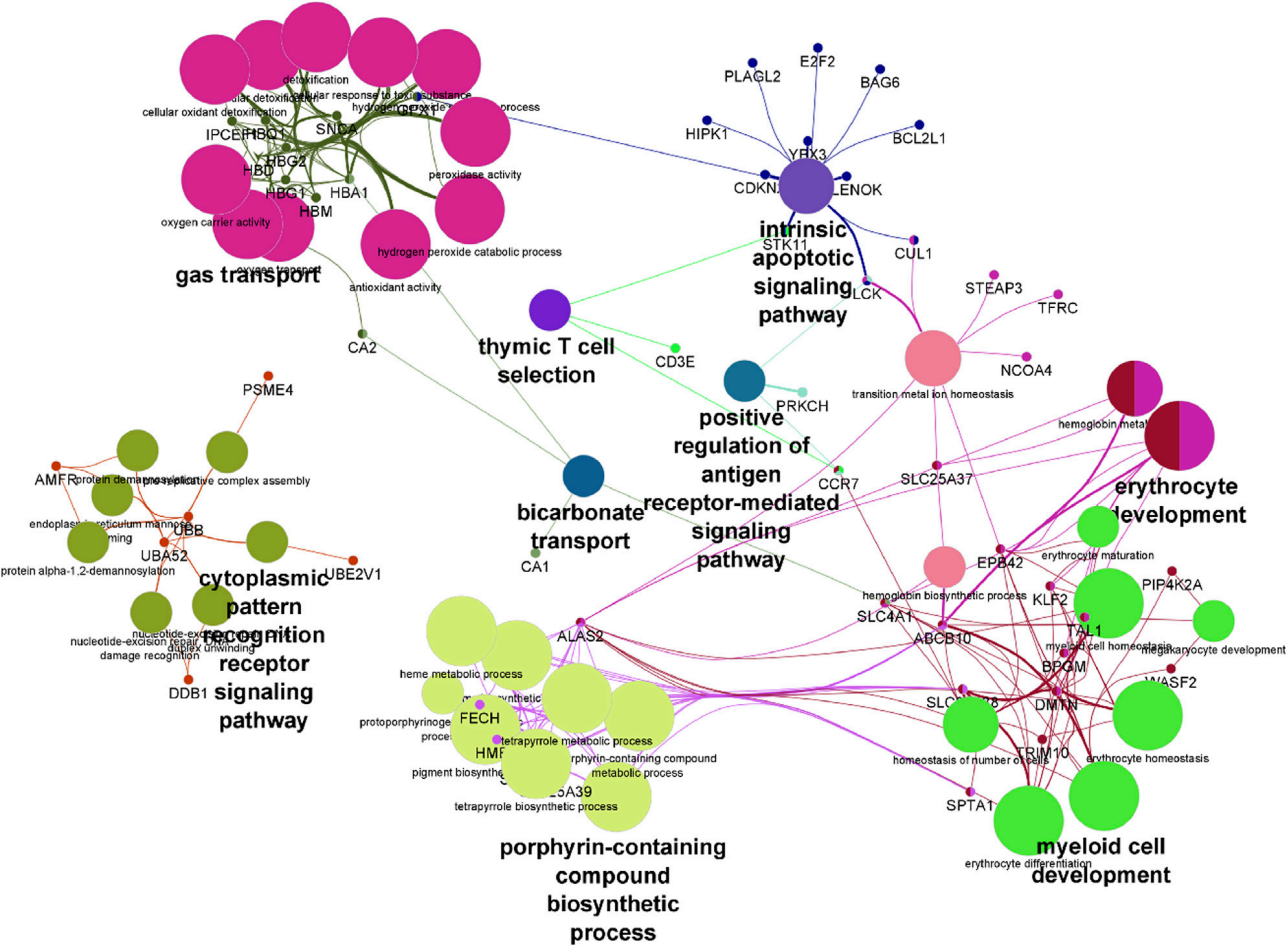
Visualization of differentially expressed genes following pathway enrichment in ClueGo. A list of differentially expressed genes were loaded into ClueGO and analyzed using the KEGG biological data base. Nodes of features are depicted by the larger shapes, and smaller circles represent individual genes.

Pathway analysis was performed on differentially expressed genes using GO enrichment module of Partek Genomics Studio. The two most prominent, most enriched pathways were associated with hemoglobin complexes and protein ubiquitination (Table2). The identification of hemoglobin is unsurprising given multiple hemoglobin family of genes showed higher RNA levels in SCA patients. In addition, pathways associated with gas transport and hemoglobin synthesis (porphyrin and tetrapyrrole synthesis) were also enriched (Table 2). Genes associated with protein ubiquitination and small molecule conjugation were enhanced in the list of differentially expressed genes, consistent with previous reports of enhanced ubiquitin proteasome system activity in SCA (Anjum et al., 2013; Warang et al., 2018). Many of these proteins were associated with E3-ligase activity and proteasome pathways, for example, SIAH2 and TRIM10. The last major grouping of enriched pathways was associated with cytoplasm and cytoskeletal structure. Results were verified using the online DAVID gene function tool, and similar results were obtained (not shown), except for the identification of trypanosomiasis and malaria disease pathways. Pathway analysis of the transcript splicing data set reveals similar hemoglobin and protein ubiquitination regulation functions (see Supplementary Material). Pathways were visualized using ClueGO (Bindea et al., 2009) (Figure 3).

**TABLE 2:** Gene Ontology categories (Top 30) for enriched pathways, based on differential gene expression analysis between SCA and Controls.

Function	Type	Enrichment score	Enrichment pvalue	% Genes in group that are present	Disease score	SCA vs. Control score	# Genes in list, in group	# Genes not in list, in group	# Genes in list, not in group	# Genes not in list, not in group	GO ID
oxygen transporter activity	molecular function	25.4421	8.92592E-12	50	5.837	5.837	7	7	149	18253	5344
oxygen transport	biological process	24.2523	2.93333E-11	43.75	5.837	5.837	7	9	149	18251	15671
porphyrin-containing compound biosynthetic process	biological process	23.873	4.28619E-11	29.6296	6.22254	6.22254	8	19	148	18241	6779
tetrapyrrole biosynthetic process	biological process	22.9251	1.10596E-10	26.6667	6.22254	6.22254	8	22	148	18238	33014
gas transport	biological process	22.3673	1.93202E-10	35	5.837	5.837	7	13	149	18247	15669
hemoglobin complex	cellular component	21.9365	2.97225E-10	50	5.95258	5.95258	6	6	150	18254	5833
porphyrin-containing compound metabolic											
Process	biological process	20.6371	1.08995E-09	20.5128	6.22254	6.22254	8	31	148	18229	6778
spectrin-associated cytoskeleton	cellular component	19.915	2.24401E-09	62.5	6.51057	6.51057	5	3	151	18257	14731
myeloid cell development	biological process	17.8016	1.85714E-08	19.4444	6.66374	6.66374	7	29	149	18231	61515
cell cortex part	cellular component	17.1603	3.52668E-08	9.00901	6.22375	6.22375	10	101	146	18159	44448
tetrapyrrole metabolic process	biological process	17.0597	3.90009E-08	13.3333	6.22254	6.22254	8	52	148	18208	33013
cofactor biosynthetic process	biological process	16.0256	1.09687E-07	8	6.16197	6.16197	10	115	146	18145	51188
protein ubiquitination	biological process	14.9672	3.16104E-07	3.36879	5.75224	5.75224	19	545	137	17715	16567
cortical cytoskeleton	cellular component	14.1374	7.2481E-07	11.6667	6.047	6.047	7	53	149	18207	30863
heme biosynthetic process	biological process	14.1086	7.45957E-07	23.8095	5.96327	5.96327	5	16	151	18244	6783
Cytosol	cellular component	14.0655	7.78838E-07	1.66889	5.83054	5.83054	50	2946	106	15314	5829
apoptotic signaling pathway	biological process	13.7862	1.02971E-06	4.1791	5.62833	5.62833	14	321	142	17939	97190
single-organism process	biological process	13.634	1.19899E-06	1.09694	5.78757	5.78757	123	11090	33	7170	44699
cofactor metabolic process	biological process	13.4312	1.46863E-06	4.39189	6.14075	6.14075	13	283	143	17977	51186
oxygen binding	molecular function	12.8207	2.70422E-06	12.766	5.95258	5.95258	6	41	150	18219	19825
protein modification by small protein conjugation	biological process	12.7395	2.93287E-06	2.90076	5.75224	5.75224	19	636	137	17624	32446
heme metabolic process	biological process	12.2238	4.91224E-06	16.6667	5.96327	5.96327	5	25	151	18235	42168
positive regulation of immune system process	biological process	11.7342	8.01482E-06	2.51196	5.7691	5.7691	21	815	135	17445	2684
protein binding	molecular function	11.6693	8.55213E-06	1.10676	5.79045	5.79045	113	10097	43	8163	5515
erythrocyte development	biological process	11.4341	1.08205E-05	23.5294	6.29449	6.29449	4	13	152	18247	48821
blood microparticle	cellular component	11.3935	1.12686E-05	6.4	5.93565	5.93565	8	117	148	18143	72562
transition metal ion homeostasis	biological process	11.2771	1.26596E-05	6.29921	5.41985	5.41985	8	119	148	18141	55076
T cell receptor signaling pathway	biological process	11.1709	1.40786E-05	7.52688	5.27692	5.27692	7	86	149	18174	50852
chemical homeostasis	biological process	11.1667	1.41374E-05	2.50313	5.70145	5.70145	20	779	136	17481	48878
signal transduction by p53 class mediator	biological process	11.1628	1.41926E-05	6.20155	5.53394	5.53394	8	121	148	18139	72331

### Regulation of Hemoglobin Genes

**FIGURE 4:**
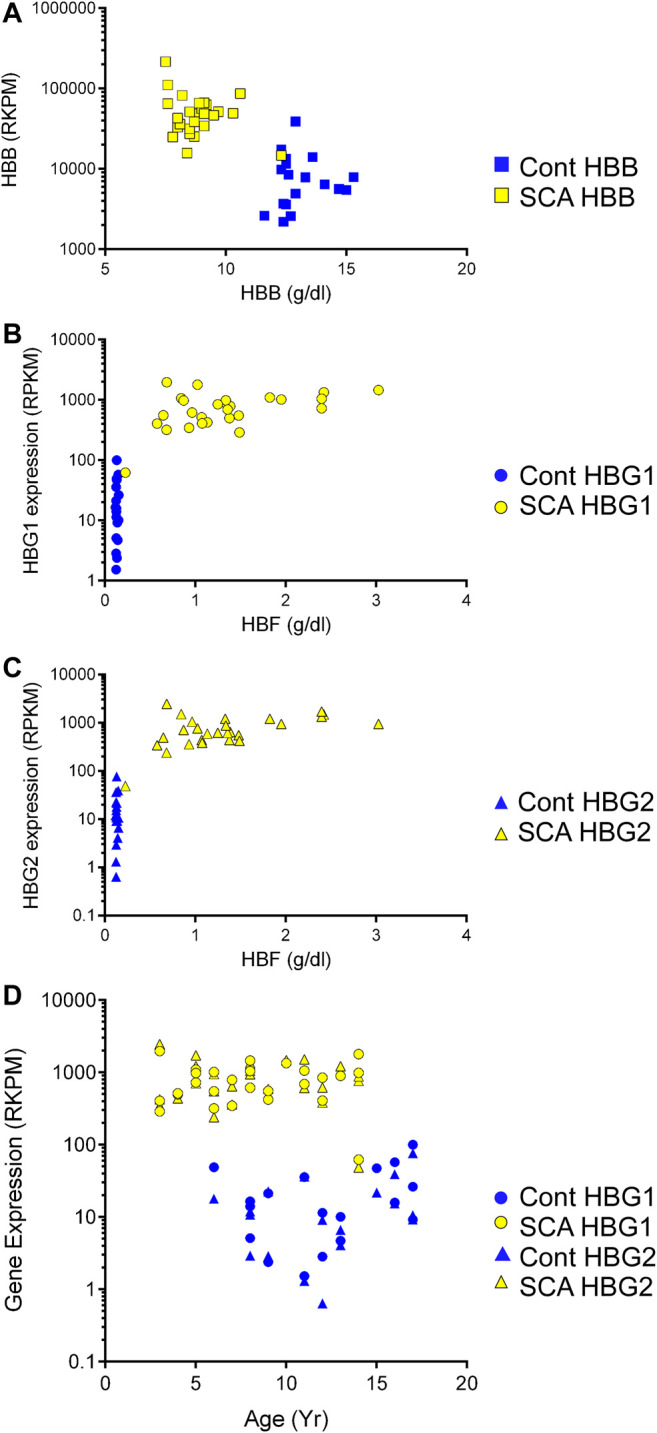
Expression of mRNA and protein of hemoglobin genes in SCA. We compared the expression of HBB, HBG1, and HBG2 with the plasma protein levels of these gene products in SCA (yellow) and controls (blue). **(A)** HBB gene expression and plasma protein levels appear inversely correlated. **(B, C)** HBG1 and HBG2 mRNA and Hbf protein levels do not correlate. **(D)** Comparison of HBG1 and HBG2 mRNA expression with participant age in controls (blue and SCA). Note the >10 fold difference in control to SCA levels of HBG1 and HBG2 gene expression values (RPKM).

We observed increased hemoglobin gene expression and alternative splicing in SCA subjects compared to controls, and oxygen transport and Hb associated pathways were regulated in GO analysis. Therefore, we asked whether RNA expression of the beta globin gene and the gamma (1 and 2) globin genes correlated to their protein levels. Protein levels of hemoglobin beta was significantly lower in SCA subjects compared to controls (Table 1, *p* < 0001). In contrast, HBB RNA levels were higher in SCA compared to controls (*p* < 0.0001 Student’s t-test) and RNA and protein levels appear inversely correlated (*r* ^2^ = 0.2239, Figure 4A).

Fetal hemoglobin (HbF) is developmentally regulated and encoded by two RNAs (HBG1 and HBG2). Fetal hemoglobin (HBF) protein levels in controls were below detection limits (< 1%). Fetal hemoglobin levels are significantly higher in SCA subjects compared to controls (*p* < 0.001, Table 1). Even though there is a range of HBF protein levels in blood of SCA subjects, the protein concentration does not show a clear linear correlation with either HBG1 or HBG2 mRNA expression levels (Figures 4B,C). When compared to subject age, HBG1 and HGB2 mRNA expression levels did not significantly correlate with age in either SCA or control subjects (Figure 4D). Together, these data show a disconnect between HBG protein levels and mRNA expression, which may be associated with cell composition of the blood, or different cells being responsible for the measured HBG protein and mRNA.

**FIGURE 5:**
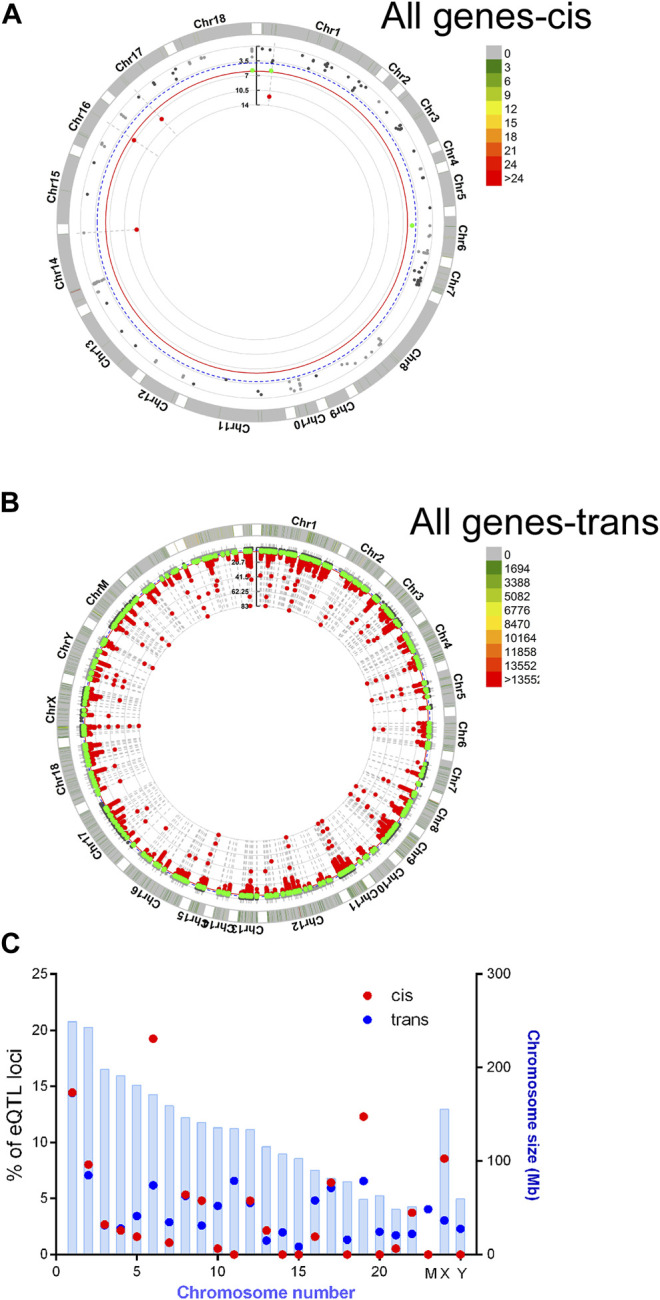
Basecalls and RPKM gene expression data were extracted using Partek and correlated using matrix eQTL package for R. Resultant data were plotted using CMplots in R (CMplot). **(A)** cis Eqtl sites **(B)** trans eQTL sites vs. entire genome expression data. **(C)** Enrichment of cis eQTLs on chr 6 and 19. The size of the chromosome in Mbp is shown in blue bars. % of identified eQTL loci/chromosome is represented by red (cis) or blue (trans) circles.

Recent human genome data suggest that single nucleotide variants may regulate gene expression and translation (GTEx Consortium, 2017; Robert and Pelletier, 2018). To find potential novel regulators of HBG we performed a targeted eQTL analysis of RNA seq data, to identify identified potential SNPS regulating HBG1 and HBG2 gene expression in SCA. Single nucleotide variations were called using Partek Genomics Studio from the RNA-Seq data, and filtered (> 20 coverage). First, basecalls were correlated to all gene expression values using matrix eQTL using disease status, age and sex as covariates (see appendix for script). Combining the data from controls and SCA together, we identified 58 and 287,111 locations potential trans and cis eQTL events (passing post-hoc FDR *p* < 0.05). The chromosomal locations of these cis and trans SNVs are represented in CMplots in Figures 5A–C.

**FIGURE 6:**
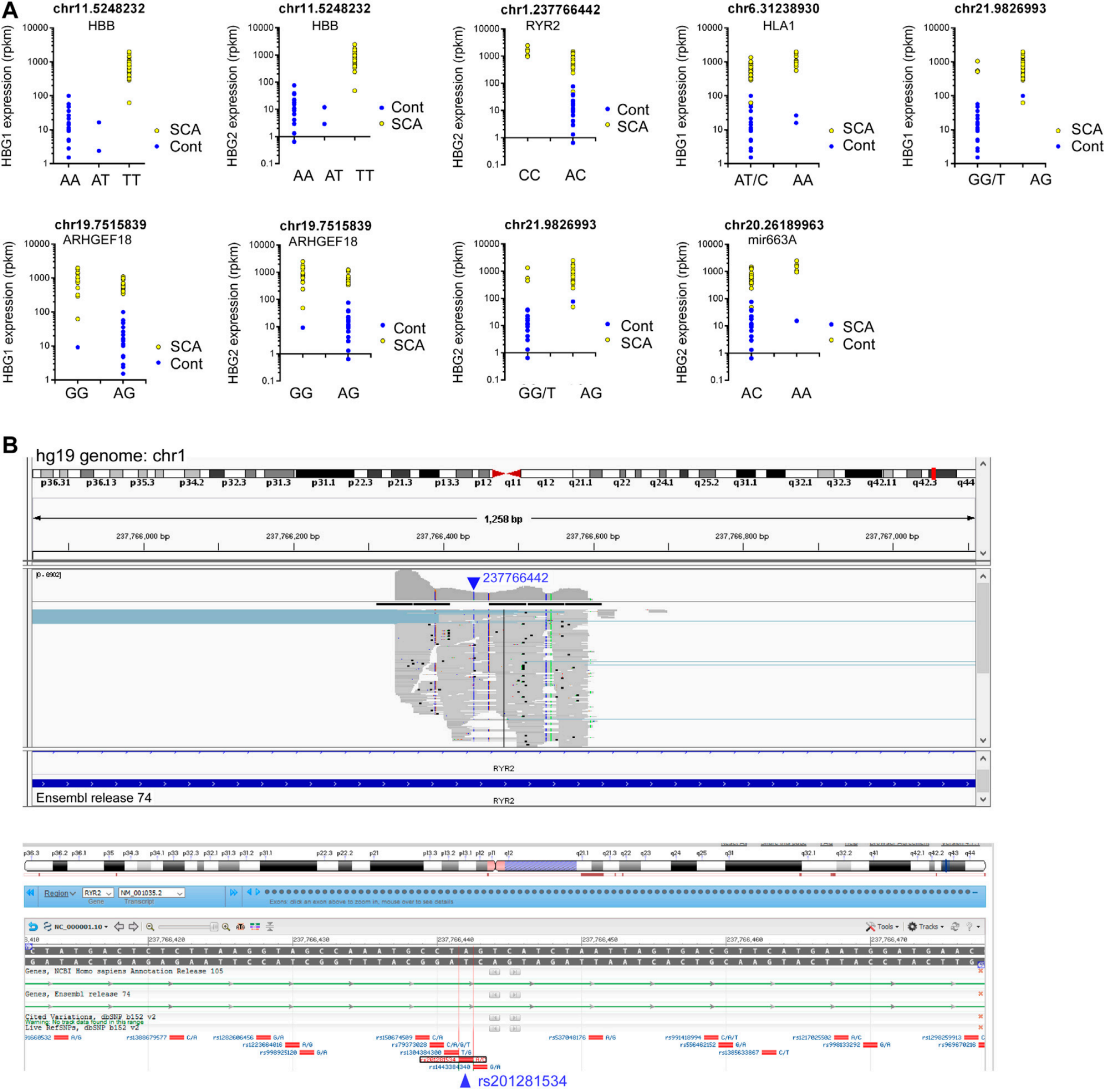
eQTL analysis of SCA RNA-Seq data reveals novel RNAs regulating HBG1 and HBG2 gene expression. **(A)** Nine loci correlated significantly with HBG1 or HBG2 gene expression (FDR adjusted *p* < 0.05). Genotype is depicted in the *x* axis, and HBG1 or HBG2 gene expression on the *Y* axis. Data show control (blue) and SCA patients (yellow). **(B)** Identification of novel intronic RNA from RYR2 visualized using the integrated genome browser (Broad Institute). The Blue arrow depicts the location of 23776642 on chr1. The gray bars represent the pile-up of reads aligning with this region. The annotation guide from ensemble release 74, depicts the region as part of an intron of RYR2. Below shows the NCBI browser screen shot depicting know SNPs in this region. The location 237766442 aligns with rs201281534 in bdSNP 152v2.

We then investigated whether any SNPs correlated with the expression of HBG1 and HBG2. We performed matrix eQTL using disease status, age and sex as covariates. No cis eQTLs significantly mapped to either HBG1 or HBG2. In contrast there were 9 trans eQTLs that correlate with HBG1 (4) and HBG2 (5) gene expression patterns, and three of these overlap (Table 3) (FDR corrected *p* < 0.05). The call of the SNVs is shown in Figure 5D using the genomic coordinates, we identify that these SNVs are all known SNVs (i.e., they are contained in clinVar/dbSNP), and associate with HBB, RYR2, HLA1, ARHGEF18, and Mir663A (Figure 5D). The HBB result (hemoglobin S mutation) is consistent with higher HbF levels in SCA. Interestingly, many of the other loci were intronic or intergenic RNAs (Table 3). We highlight one intronic eQTL loci that appears to be a novel non-coding RNA contained within the intron of the RYR2 gene (Figure 6B), and the loci is a known SNV (dbSNP 155v2; hg19; rs201281534) (Figure 6B).

**TABLE 3:** *Trans*-SNPs identified in Matrix eQTL as correlating with HBG1 or HBG2 expression.

SNP	Gene	Beta	*t*-stat	*p*-value	FDR	dbSNP_151	Gene	Location
chr11.5248232	HBG1	417.2	5.9930	4.43E-07	0.00068	rs334	HBB	exon
chr11.5248232	HBG2	419.4	5.3095	4.12E-06	0.00393	rs334	HBB	exon
chr1.237766442	HBG2	−1062.6	−4.9667	1.25E-05	0.00902	rs201281534	RYR2	intron
chr6.31238930	HBG1	671.7	4.9351	1.38E-05	0.00976	rs2308592	HLA_1	exon
chr21.9826993	HBG1	625.1	4.7840	2.24E-05	0.01389	rs1297551451		intergenic
chr19.7515839	HBG1	−616.5	−4.6012	4.00E-05	0.02111	rs372840184	ARHGEF18	intron
chr19.7515839	HBG2	−650.9	−4.4713	6.03E-05	0.02815	rs372840184	ARHGEF18	intron
chr21.9826993	HBG2	630.1	4.3350	9.23E-05	0.03783	rs1297551451		intergenic
chr20.26189963	HBG2	810.3	4.2602	1.16E-04	0.04454	RS126185226	mir663A	exon *

Finally, we repeated out analysis using just the splitting the SCA and control data into two individual analyses, to overcome the potential effect of using HBG1/2 gene expression as a surrogate for SCA status. Matrix eQTL analysis using age and sex as covariates did not yield any significant trans or cis eQTLs. These data suggest that SNPs associated with non-coding, or novel RNAs may play a role in either HBG1/HBG2 gene expression, but may also correlate to SCA status of a patient, and therefore deserve further investigation.

**FIGURE 7:**
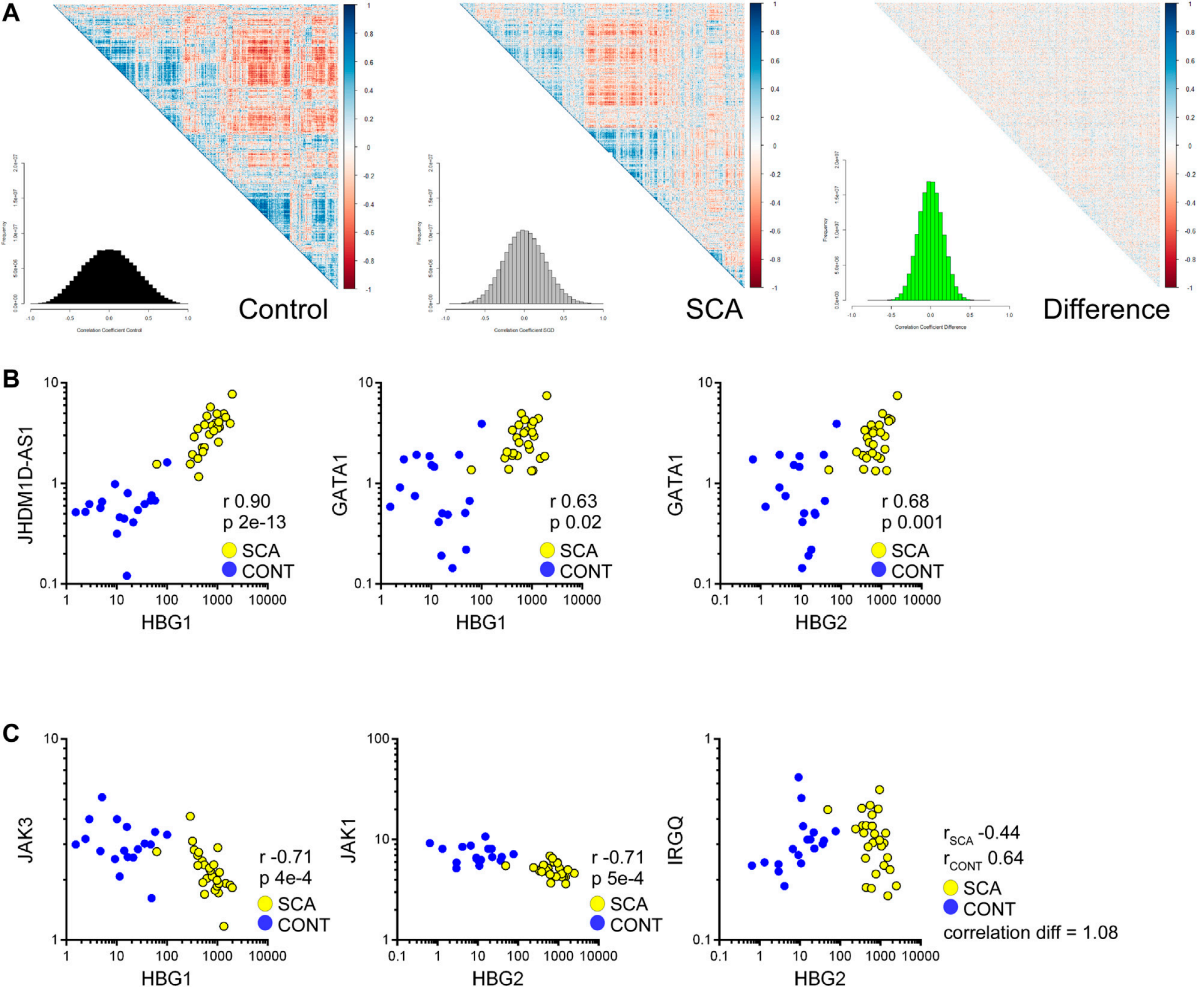
Correlation analysis of RNA-Seq data reveals reduced coordination in SCA and identifies pathways regulating HBG1 and HBG2 expression. **(A)** Heatmap of correlation matrices of gene expression values in control and SCA. The difference was normalized to 1 and also plotted. The frequency of correlation values was determined using the histogram package, and plotted as inset. Note the reduced correlation in the SCA data. **(B)** Correlation between HBG1 and HBG2 with JHDM1D-AS1 and GATA1 across all data sets: Control (blue) and SCA(yellow). Correlation r values and *p* values (Bonferroni corrected) are shown inset. **(C)** Inverse correlation of HBG1 with Jak3 and HBG2 with Jak1. IRGQ showed the greatest change in correlation between the two patient groups, changing from a positive correlation in Controls to a negative correlation in SCA (yellow) subjects. Spearman Correlation performed in Partek Genomics Studio, all *p*-values were *p* < 0.05 following Bonferroni correction for multiple testing.

We further investigated our data set for genes whose expression may correlate with HBG genes, to identify potential regulatory elements/factors. Correlation analysis was performed to determine whether genes associated with HBG1 or HBG2 mRNA expression were regulated in SCA. SCA and controls data were subjected to Spearman correlation (to reduce the influence of outliers) and correlation coefficients were grouped using hierarchical clustering (Figures 6A,B: The distributions of correlation coefficients is shown in a histogram inset). The control data appears more co-regulated, as determined by clusters within the heatmap, with a trend towards lower correlation values in the SCA data set. The difference between the control and SCA data was also determined, from this analysis we do not observe a strong-coordinated correlation shift between the data (most data are distributed around 0: Figure 7C inset). These data suggest that gene expression is less correlated in SCA.

We prepared a list of genes whose correlation changes by 0.5 and subjected the lists to pathway analysis. The most differentially regulated was IRGQ (Figure 7E) with a correlation change of 1.0 (from 0.63 to 0.44 controls to SCA). Genes that show a reduced correlation in SCA compared to control are enriched for transcription and translation regulation pathways (Supplementary Material). In contrast, no obvious key mechanisms appeared to be overrepresented in the increased correlation data set (Supplementary Material). This suggests that although RNA expression is increased overall in SCA, it is less coordinated with respect to biological pathways.

From these data, we searched for genes that correlate with γ-globin genes, specifically potential transcription or translation regulating genes, and microRNAs associated with these regulators, or with direct correlation to the hemoglobin genes. We found 1381 genes expression values correlated with either HBG1 or HBG2 (Bonferroni corrected *p*-value *p* < 0.05). From these we subjected the gene lists to Gene Ontology analysis. When we split the data into SCA or control we observe the positively correlated genes associated with protein ubiquitination associated pathways, including E2 and E3 ligases (Supplementary Material). We also identified within this list GATA1 expression levels correlating with HBG1 and HBG2 expression levels (Figure 6B), suggesting an increased GATA 1 drive associated with higher expression of fetal hemoglobin genes. In contrast, the negatively associated genes appear to involve immune responses to interleukins, and specifically members of the JAK-STAT signaling pathway (Supplementary Material: Figure 7B). JAK inhibitors have recently been investigated as potential therapies for sickle cell disease (Pecoraro et al., 2015). Again, whilst these correlations appear provocative, further follow study is required.

## Discussion

In this study, we performed whole transcriptome analysis of peripheral blood in children with sickle cell anemia and identified differential gene and transcript expression in SCA. We also identified potential eQTL loci which may be responsible for higher γ-globin RNA expression in SCA subjects, and potential novel regulators of HBG1 and HBG2 RNA expression. While this is a relatively small study, our observations regarding the overall transcriptomic effect of sickle cell anemia appear consistent with multiple previous reports. Children with sickle cell anemia show fundamental changes in their blood transcriptome, with differential mapping, and approximately 8% of the detected transcriptome showing differential expression (Figure 2). This suggests that some of the pathophysiologic responses of SCA may be due to this considerable change in transcription. Furthermore, the disorder appears to de-regulate the co-expression networks of multiple genes. Similar to other published blood transcriptome studies in adults (see below), we observe changes in hemoglobin expression, and enhanced expression of proteins associated with protein degradation (ubiquitin proteasome system), cytoskeletal compensation, and autophagy/mitophagy. This last biological pathway is currently under investigation to reduce the mitochondria content of red blood cells (Archer et al., 2015).

We observe both genes encoding γ-globin (HBG1 and HBG2) are increased in SCA compared to controls, and the SCA subjects in this study have higher HbF protein levels compared to controls. To avoid treatment effects, we recruited SCA subjects who were not receiving chronic red cell transfusions or hydroxyurea; this approach selects for less symptomatic subjects who may have higher Hb F levels. It is of note that hydroxyurea increases both mRNA and protein levels two fold (Italia et al., 2013), whereas the absolute difference between HBG1 and HBG2 mRNAs in SCA subjects vs. controls in our study is in the order of 48 and 70 fold, respectively.

Multiple transcriptional regulatory elements enhance or inhibit the globin gene loci. Expression of known drivers of HBG1 and HBG2 genes, such as KLF10 and SIRT1 were not significantly increased in our samples (not shown) (Borg et al., 2012; Dai et al., 2017). We also did not find any significant differences in expression of the common inhibitory regulators of HBG1 and HBG2 gene [KLF1 (Zhou et al., 2010; Grieco et al., 2015; Vinjamur et al., 2016), ALAS1, Bcl11A, HGC1, and BGLT3 (Ivaldi et al., 2018)]. Furthermore, there was no correlation between Hb F protein levels and either HBG1 or HBG2 RNA expression levels. This suggests that the mechanisms driving HBG1 and HBG2 mRNA expression in whole blood samples appear different from those driving red blood cell γ-globin translation and protein expression. Recent studies have attempted to regulate HbF at the translational level (Hahn and Lowrey, 2014). Together, this mismatch between γ-globin gene and protein expression suggests that yet unidentified factors may contribute to HbF transcription and translation and these could make novel therapeutic targets.

In order to identify potential regulators of HBG1 and HBG2 RNA expression, we performed both eQTL and expression correlation investigations. SNPs in the promotor of these genes were associated with HbF expression in a Brazilian SCA study (Barbosa et al., 2010). In our study, we did not identify any cis (local) SNPs associated with differential HBG1 or HBG2 expression. We found some trans SNPs that correlate with HBG1/HBG2 expression. These RNAs appear to be novel non-coding regulatory RNAs, and were not found in the current human genome annotation guides (ensembl), however all of the SNVs were found in the current SNP database (dbSNP v 152). The identification of these novel RNAs was only possible with whole transcriptome RNA-seq methodology, and offer the potential for novel approaches to regulate fetal hemogobin expression.

We performed correlation analysis on controls and SCA datasets to identify gene relationships within our dataset, and potential regulators of HBG1 and HBG2. Such an approach has not yet been performed on SCA blood data sets. HBG1 showed a highest correlation with JHDM1D-As1, a long-noncoding RNA associated with angiogenesis (Kondo et al., 2017). GATA 1, a driver of globin gene expression (Testa, 2009), was modestly positively correlated with HBG1 and HBG2 in SCA compared to control. Correlation analysis of HBG1 and HBG2 with the rest of the transcriptome reveals a negative correlation with JAK-STAT signaling pathway members (JAK1 and JAK2), which is consistent with reports identifying enhanced fetal γ-globin levels in cells treated by JAK stat inhibitors (Pecoraro et al., 2015). This suggests both a loss of repression and a promoting effect of GATA1 may drive the increase in HBG gene expression in SCA, which could be further investigated for therapeutic synergy.

### Comparison to Previous Transcriptome Studies

Previous microarray studies in SCA using platelets, PBMCs, and whole blood reveal similar patterns as those reported here (Desai et al., 2017; Raghavachari et al., 2009; Raghavachari et al., 2007; Raghavachari et al., 2012; Hounkpe et al., 2015). An increase in platelet RNA expression is observed in SCA patients (220 genes were identified as DE, FC ± 5.0, FDR < 0.2) compared to controls (Raghavachari et al., 2007). We observe multiple platelet associated RNAs in our whole blood RNA-seq data (for example, PPBP, PF4, and NRGN), suggesting a component of whole blood RNA signal is derived from platelets. RNA expression in adult SCA whole blood cells was analyzed using microarray [compare Figure 2A this study vs. Figure 6A (Raghavachari et al., 2009)]. They identified 112 genes with differential expression (FC ± 2, FDR < 0.2), and in particular multiple hemoglobin genes showing increased expression in SCA (including HBM, HBG, and HBBP1) (Raghavachari et al., 2009). The same group showed RNA-Seq identifies novel transcripts and differential expression in SCA, although this study had lower depth of sequencing than our study, and only investigated four samples. In general, microarray responses show a smaller fold change compared to RNA-Seq studies (Raghavachari et al., 2012). Our study identified more differentially expressed genes, probably due to the larger number of samples in our study, and second due to our study using whole transcriptome analysis with a newer reference, versus polyA transcripts (Raghavachari et al., 2012).

A recent meta-analysis of sickle cell disease gene expression data sets has been performed on previously reported microarray data (Hounkpe et al., 2015). One of these studies (GEO accession number GEO35007) investigated blood RNA expression in children with SCA, both in acute crisis and steady state. The meta-analysis identified similar pathways of enriched genes following differential expression analysis, including immune response, autophagy, stress, heme metabolism, and synthesis (Hounkpe et al., 2015), consistent with our observations. Meta-analysis also confirms a general trend of increased gene expression in SCA vs. controls. Interestingly, a recent study applied transcriptomic findings to enhance clinical diagnosis (mortality prediction) in SCA (Desai et al., 2017).

### Limitations

We acknowledge that our study has a small sample size (due to patient cohort size), which may affect the power of some observations. To correct for this all data were subjected to post hoc correction. We did observe significant gene expression and transcript usage changes in SCA vs. controls. We did not perform validation of our expression results with PCR because we noticed strong consistency with similar studies (Raghavachari et al., 2007; Raghavachari et al., 2009; Raghavachari et al., 2012; Hounkpe et al., 2015; Desai et al., 2017).

In summary, while we identified some patterns of differential gene expression observed in prior microarray and RNAseq studies, our study is distinguished by several features. By limiting our study to children not receiving HU or red cell transfusion therapy, this group with higher baseline hemoglobin F levels enabled discovery of potential novel γ-globin regulators. The use of whole transcriptome, non-ribosome/globin depleted RNA enables the identification of novel RNAs and the correlation to hemoglobin genes. We chose not to remove globin mRNA from our samples based on our experiences of ribosomal reduction techniques in PAXgene tube derived RNA, and the observations of others (Liu et al., 2006; Raghavachari et al., 2012). This permitted discovery of red cell gene expression while also identifying many of the genes revealed in globin depleted microarray studies, suggesting a potential advantage of RNA-seq methodology without globin mRNA depletion. Other sickle cell transcriptome studies have not previously applied eQTL and gene expression correlation analysis. These methods confirmed known γ-globin regulators, GATA 1 and JAK 1 and JAK 2, and identified two non-coding sequences strongly associated with HBG expression. Lastly, the lack of association between HBG1 and HGB2 gene and Hemoglobin F protein expression suggest that there is post-translational regulation of fetal hemoglobin synthesis, potentially mediated by the upregulated ubiquitin-proteasome pathways. Taken together, these data show the depth of biological information to be extracted from RNA-sequencing studies of SCA patients.

## Data Availability

Data are available at dbGAP, Accession: phs002687.v1.p1 (http://www.ncbi.nlm.nih.gov/projects/gap/cgi-bin/study.cgi?study_id=phs002687.v1.p1). Further queries can be directed to the corresponding author.
