# Supplementary material for: Whole Blood Transcriptome Analysis in Children with Sickle Cell Anemia
Source: Front Genet. 2022 Jan 13;12:737741. doi: 10.3389/fgene.2021.737741 (PMC8793691; doi:10.3389/fgene.2021.737741)
Supplement: Supplementary file 2 [file Table1.DOCX]

R Script for matrix eQTL

###########################snpReady#################

#https://cran.r-project.org/web/packages/snpReady/snpReady.pdf

#setup and clean up

rm=(list(ls))

setwd("path/to/working/directory")

getwd()

#Load packages

library(snpReady)

library(MatrixEQTL)

library(CMplot)

# Set Data directory and results directory

DATA="path/to/EQTL_PROJECT/DATA"

RESULTS="path/to/EQTL_PROJECT/Results"

PLOTS="path/to/EQTL_PROJECT/Images"

## 1- Load library and Data

string.as.factors=FALSE

data <- read.table(file.path(DATA,"BaseCalls_SNPS.txt"), sep = "\t", header=TRUE, na.strings = "NN")

dim(data)

data[1:10,1:20]

snp <- data[, -(1:2)]

rownames(snp) <- data [,2]

snp <- t(snp)

snp[1:10,1:10]

# Clean data and recode

geno.ready <- raw.data(data = as.matrix(snp), frame = "wide", base = TRUE, sweep.sample = 0.5, call.rate = 0.90, maf = 0.05, imput = TRUE, imput.type = "wright", outfile = "012")

M <- geno.ready$M.clean

M[1:10, 1:10]

geno.ready$report

cleaned <-(as.data.frame(M))

cleaned[1:10,1:10]

dim(cleaned)

head(cleaned)

# and save

write.table(cleaned, file = file.path(RESULTS,"cleanedBaseCalls_SNPS.txt"), sep = "\t")

## 2 - Prepare covariates as numeric

#load data file, rename rows and then transpose

cdata <- read.table(file.path(DATA,"Covariates.txt"), sep = "\t", header = TRUE)

head(data)

#label rows with ID

cov <- cdata[,-1]

rownames (cov) <- cdata[,1]

head (cov)

#only keep age and sex columns to remove disease associated covariate

dat <- cov[,c(3,6,7)]

head(dat)

str(dat)

dat$DISEASE <- as.numeric(factor(dat$DISEASE, levels=c("Control", "SCA")))

dat$Sex <- as.numeric(factor(dat$Sex, levels=c("Male", "Female", "Unknown" )))

str(dat)

head(dat)

dat <- t(dat)

dim(dat)

# and save

write.table(dat, file = file.path(RESULTS,"Numeric_Covariate2.txt"), sep="\t")

## 3 - Import genpos data and remove last column

gdata <- read.table(file.path(DATA,"genepos_gene.txt"), sep = "\t", header = TRUE)

head(gdata)

datag <- gdata[,-5]

write.table(datag, file = file.path(RESULTS,"genepos.txt"), sep = "\t")

#Clean GENE expression data

data <- read.table(file.path(DATA,"GENE.txt"), sep = "\t", header = TRUE)

head(data)

dim(data)

data[1:20,1:20]

## analysis of just HBG genes

gen <- cbind(data$HBG1, data$HBG2)

colnames(gen) <- c("HBG1", "HBG2")

rownames(gen) <- data[,1]

gene <-t(gen)

head(gene)

dim (gene)

rows <- match(colnames(cleaned), colnames(gene))

gen2 <- gene[,rows]

dim(gen2)

# and save

write.table(gen2, file = file.path(RESULTS,"GENEHBG.txt"), sep = "\t")

### 4 Now use MatrixEQTL

#http://www.bios.unc.edu/research/genomic_software/Matrix_eQTL/runit.html#own

rm = list(ls())

# set file directory and model

DATA="path/to/EQTL_PROJECT/DATA"

RESULTS="path/to/EQTL_PROJECT/Results"

PLOTS="path/to/EQTL_PROJECT/Images"

base.dir= ("path/to/EQTL_PROJECT/Results")

#now load data files

SNP_file_name = paste(base.dir, "/cleanedBaseCalls_SNPS.txt", sep="")

expression_file_name = paste(base.dir, "/GENEHBG.txt", sep="");

covariates_file_name = paste(base.dir, "/Numeric_Covariate2.txt", sep="");

SNP_position_file_name = file.path(DATA, "snpspos2.txt", sep="");

gene_position_file_name = paste(base.dir, "/genepos.txt", sep="");

snpspos <- read.table(SNP_position_file_name, sep = "\t", header = TRUE)

genepos <- read.table(gene_position_file_name, sep = "\t", header = TRUE)

# set Statistical threashold 0.01

useModel = modelLINEAR;

pvOutputThreshold_cis = 1e-3;

pvOutputThreshold_tra = 1e-3;

errorCovariance = numeric();

cisDist= 1e6

output_file_name_cis = "SCDcis2.txt";

output_file_name_tra = "SCDtra2.txt";

## Load genotype data

snps = SlicedData$new();

snps$fileDelimiter = "\t"; # the TAB character

snps$fileOmitCharacters = "NA"; # denote missing values;

snps$fileSkipRows = 1; # one row of column labels

snps$fileSkipColumns = 1; # one column of row labels

snps$fileSliceSize = 2000; # read file in slices of 2,000 rows

snps$LoadFile(SNP_file_name);

## Load gene expression data

gene = SlicedData$new();

gene$fileDelimiter = "\t"; # the TAB character

gene$fileOmitCharacters = "NA"; # denote missing values;

gene$fileSkipRows = 1; # one row of column labels

gene$fileSkipColumns = 1; # one column of row labels

gene$fileSliceSize = 2000; # read file in slices of 2,000 rows

gene$LoadFile(expression_file_name);

## Load covariates

cvrt = SlicedData$new();

cvrt$fileDelimiter = "\t"; # the TAB character

cvrt$fileOmitCharacters = "NA"; # denote missing values;

cvrt$fileSkipRows = 1; # one row of column labels

cvrt$fileSkipColumns = 1; # one column of row labels

if(length(covariates_file_name)>0) {

cvrt$LoadFile(covariates_file_name);

}

## Finally, the main Matrix eQTL function is called:

me = Matrix_eQTL_main(

snps = snps,

gene = gene,

cvrt = cvrt,

output_file_name = output_file_name_tra,

pvOutputThreshold = pvOutputThreshold_tra,

useModel = useModel,

errorCovariance = errorCovariance,

verbose = TRUE,

output_file_name.cis = output_file_name_cis,

pvOutputThreshold.cis = pvOutputThreshold_cis,

snpspos = snpspos,

genepos = genepos,

cisDist = cisDist,

#pvalue.hist = TRUE,

pvalue.hist = "qqplot",

min.pv.by.genesnp = FALSE,

noFDRsaveMemory = FALSE);

# this stops the results file being written

#unlink(output_file_name_tra);

#unlink(output_file_name_cis);

#cat('Analysis done in: ', me$time.in.sec, ' seconds', '\n');

#cat('Detected eQTLs:', '\n');

#show(me$all$eqtls)

cat('Analysis done in:', me$time.in.sec,'seconds','\n');

cat('Detected local eQTLs:','\n');

show(me$cis$eqtls)

cat('Detected distant eQTLs:','\n');

show(me$trans$eqtls)

#depending on pvalue setting either histogram or qqplot is drawn.

plot (me)

#### 5- preparing the results for using CMplot

#It would be nice to match and rename the snp data by rs numbers....

library(dplyr)

library(tidyr)

head(me$cis$eqtls)

head(me$trans$eqtls)

# copy column to cis data

data_cis <- (me$cis$eqtls)

data_cis$chr = data_cis$snps

dim(data_cis)

# copy column in trans

data_trans <- (me$trans$eqtls)

data_trans$chr = data_trans$snps

dim(data_trans)

#method 1 to split column _USE THIS to split columns and extract numbers. may need to change to numeric.

data2 <- (data_cis %>% separate(chr, into = c("CHR", "BP"), sep = "([\\.])", convert = TRUE))

data2$num = data2$CHR

head(data2)

cis <- data2 %>% separate(num, into = c("num", "num2"), sep = "([\\chr\\h\\r])", convert = TRUE)

dim(cis)

cisdata <- cis[order(cis$num2),]

head(cisdata)

write.table(cisdata, file=file.path(RESULTS, "result_for_CMplot_cis_2.txt"), sep = "\t")

data5 <- (data_trans %>% separate(chr, into = c("CHR", "BP"), sep = "([\\.])", convert = TRUE))

data5$num = data5$CHR

head(data5)

trans <- data5 %>% separate(num, into = c("num", "num2"), sep = "([\\chr\\h\\r])", convert = TRUE)

transdata <- trans[order(trans$num2),]

head(transdata); dim(transdata)

write.table(transdata, file=file.path(RESULTS,"result_for_CMplot_trans_2.txt"), sep = "\t")

##6. CMplots

result_C <- read.table(file.path(RESULTS, "result_for_CMplot_cis.txt"), sep = "\t", header = TRUE)

result_T <- read.table(file.path(RESULTS, "result_for_CMplot_trans_2.txt"), sep = "\t", header = TRUE)

dim(result_C)

dim(result_T)

resultC <- result_C[ ,c(1, 7, 8, 5)]

names(resultC) <- c("SNP", "CHR", "BP", "P")

head(resultC)

resultT <- result_T[ ,c(1, 7, 8, 5)]

names(resultT) <- c("SNP", "CHR", "BP", "P")

head(resultT)

resultT

# how many snps per chr?

write.table((as.data.frame(table(resultC$CHR))), file=file.path(RESULTS,"result_cis_per_chr.txt"), sep = "\t")

write.table((as.data.frame(table(resultT$CHR))), file=file.path(RESULTS,"result_trans_per_chr.txt"), sep = "\t")

## plot cis

# SNP.density plot

CMplot(resultC,plot.type="d",bin.size=1e6,col=c("darkgreen", "yellow", "red"),file="jpg",memo="cis",dpi=600,

file.output=TRUE)

#circular plot with extras- better

CMplot(resultC,plot.type="c",r=3,col=c("grey30","grey60"),chr.labels=paste("Chr",c(1:18,"X","Y","M"),sep=""),

threshold=c(1e-6,1e-4),cir.chr.h=1.5,amplify=TRUE,threshold.lty=c(1,2),threshold.col=c("red",

"blue"),signal.line=1,signal.col=c("red","green"),chr.den.col=c("darkgreen","yellow","red"),

bin.size=1e6,outward=FALSE,file="jpg",memo="cis",dpi=600,file.output=TRUE)

CMplot(resultC, plot.type="m", col=c("grey30","grey60"), LOG10=TRUE, ylim=c(2,60), threshold=c(1e-6,1e-4),

threshold.lty=c(1,2), threshold.lwd=c(1,1), threshold.col=c("black","grey"), amplify=TRUE,

chr.den.col=NULL, signal.col=c("red","green"), signal.cex=c(1,1),signal.pch=c(19,19),

file="jpg",memo="cis",dpi=600,file.output=TRUE)

## now plot trans

# SNP.density plot

CMplot(resultT,plot.type="d",bin.size=1e6,col=c("darkgreen", "yellow", "red"),file="jpg",memo="tra",dpi=600,

file.output=TRUE)

#circular plot with extras- better

CMplot(resultT,plot.type="c",r=3,col=c("grey30","grey60"),chr.labels=paste("Chr",c(1:18,"X","Y","M"),sep=""),

threshold=c(1e-6,1e-4),cir.chr.h=1.5,amplify=TRUE,threshold.lty=c(1,2),threshold.col=c("red",

"blue"),signal.line=1,signal.col=c("red","green"),chr.den.col=c("darkgreen","yellow","red"),

bin.size=1e6,outward=FALSE,file="jpg",memo="tra",dpi=600,file.output=TRUE)

#circular plot with extras- better

CMplot(resultT,plot.type="c",r=3,col=c("grey30","grey60"),chr.labels=unique(resultT$CHR),

threshold=(0.05),cir.chr.h=1.5,amplify=TRUE,threshold.lty=c(1,2),threshold.col=c("red",

"blue"),signal.line=1,signal.col=c("red","green"),chr.den.col=c("darkgreen","yellow","red"),

bin.size=1e6,outward=FALSE,file="jpg",memo="tra",dpi=600,file.output=TRUE)

CMplot(resultT, plot.type="m", col=c("grey30","grey60"), LOG10=TRUE, ylim=c(2,60), threshold=c(1e-6,1e-4),

threshold.lty=c(1,2), threshold.lwd=c(1,1), threshold.col=c("black","grey"), amplify=TRUE,

chr.den.col=NULL, signal.col=c("red","green"), signal.cex=c(1,1),signal.pch=c(19,19),

file="jpg",memo="tra",dpi=600,file.output=TRUE)
